# Acute Left-Side Colonic Diverticulitis: A Historical Cohort Study on the Optimization of Non-Operative Management Outcomes and Anastomosis Rate After Sigmoid Resection

**DOI:** 10.3390/jcm14134658

**Published:** 2025-07-01

**Authors:** Ana Isabel Fernández Sánchez, José Manuel Aranda Narváez, Irene Mirón Fernández, Julio Santoyo Santoyo

**Affiliations:** 1 Trauma and Emergency Surgery Division, General, Digestive and Transplantation Surgery Department, University Regional Hospital, 400056 Malaga, Spain; jose.aranda.narvaez@gmail.com; 2 Faculty of Medicine, Malaga University, 29071 Malaga, Spain; julio.santoyo.sspa@juntadeandalucia.es; 3 General, Digestive and Transplantation Surgery Department, University Regional Hospital, 400056 Malaga, Spain

**Keywords:** diverticulitis, conservative treatment, sigmoid colectomy, anastomosis, colostomy

## Abstract

**Background**: The guidelines proposed by the World Society for Emergency Surgery (WSES) in 2020 for the management of Acute Diverticulitis (AD) emphasize the safety of non-operative management (NOM) in selected patients and recommend sigmoid resection (SR) with primary anastomosis (PA) as the surgical technique of choice. Aim: To analyze the clinical outcomes following the implementation of an evidence-based clinical pathway developed by an Acute Care Surgery Unit (ACSu) at a reference center. Methods: For analysis, patients were divided into two groups: pre-guidelines (2018–2019) and post-guidelines (2020–2023), following the May 2020 WSES publication. Patients were classified according to the WSES classification. **Results**: NOM failure and PA after SR rates by groups were as follows (NOM failure not applicable to III–IV): 0-IA, 2.7% and 94.7%; IB–IIA, 16% and 85.2%; IIB, 7.1% and 50%; III–IV, 75.6%. The global stoma-free rate was 78.8%, with a 15.7% anastomotic leak rate and 14.2% significant morbidity, with an increased rate of anastomosis in unstable patients thanks to Damage Control Surgery (DCS). A logistic regression model was performed to identify factors associated with postoperative morbidity. Patients who underwent primary anastomosis had a lower risk of postoperative morbidity compared to those treated with Hartmann’s procedure (OR = 0.22 (0.04–1.25), *p* = 0.088). **Conclusions**: Excellent outcomes in AD healthcare may be achieved if evidence-based recommendations are followed. The ACSu plays a key role in designing and promoting these protocols.

## 1. Introduction

The incidence of Acute Diverticulitis (AD) is progressively increasing in Western countries. The economic burden on healthcare systems in both the United States and Europe is significant; therefore, efforts to optimize AD management should be strongly considered [1,2,3].

Several guidelines summarize evidence-based recommendations for the appropriate diagnosis and treatment of AD. Once AD is suspected, a CT scan must be performed to establish a diagnosis and determine the severity grade [4]. Uncomplicated and early-stage complicated AD in healthy patients can be managed with NOM, with or without antibiotics, in an outpatient setting. Other patients require hospitalization for antibiotic therapy, which should be adjusted according to local protocols based on the most frequently isolated bacteria. Percutaneous drainage (PD) should be considered in cases with large, accessible abscesses. When surgical intervention is necessary, sigmoid resection (SR) is the most widely accepted technique, preferably performed laparoscopically if adequate equipment and expertise are available [1,2]. For hemodynamically stable patients, the recommended approach following SR is primary anastomosis (PA), with or without a defunctioning ileostomy (PI), while Hartmann’s Procedure (HP) should be reserved for frail or unstable patients [5,6]. Damage Control Surgery (DCS), involving a rapid SR and temporary abdominal closure (TAC) using a vacuum-assisted device, should be considered in cases of severe physiological impairment, as improved clinical conditions in a subsequent procedure may allow for a delayed anastomosis [7].

Concerns about the postoperative course of emergency colorectal anastomosis and the application of DCS principles in septic abdomen raise doubts among surgeons managing AD [8,9].

This study aimed to analyze the clinical outcomes achieved by strictly following clinical guidelines, based on data collected over a six-year period, and to evaluate whether these outcomes improved following the implementation of an evidence-based hospital protocol.

## 2. Materials and Methods

**Figure 1:**
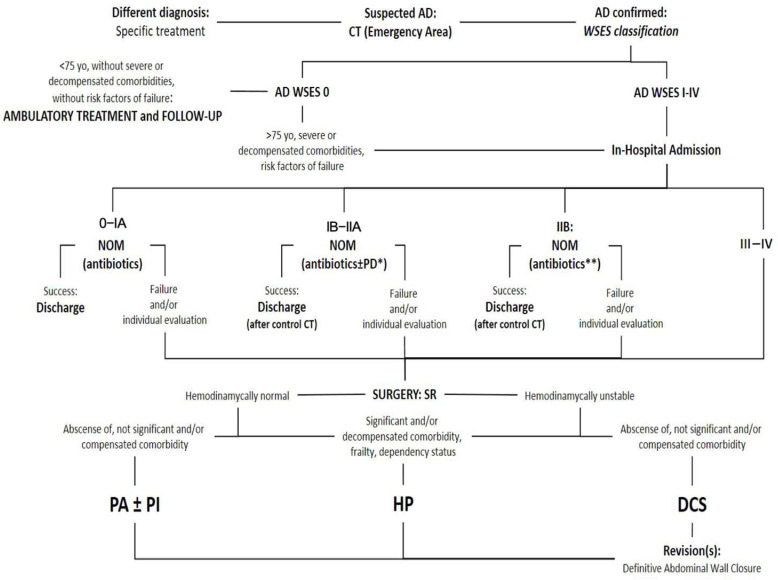
Resumed protocol for Acute Diverticulitis healthcare from the University Regional Hospital of Malaga, Spain. AD: Acute Diverticulitis. CT: Computed Tomography. WSES: World Society of Emergency Surgery. NOM: Non-operative management. PD: Percutaneous drainage. *: Accessible abscess > 4 cm: systematic drainage; specific consideration of <4 cm and non-accessible abscesses. **: Only for hemodynamically normal patients and without clinical signs of diffuse peritonitis. SR: sigmoid resection. PA: primary anastomosis. PI: defunctioning proximal ileostomy. HP: Hartmann’s procedure. DCS: Damage Control Surgery.

The University General Hospital is the referral center of Malaga (Spain). The General, Digestive, and Transplantation Surgery Department has 31 staff surgeons and 10 residents attached, with 2 staff and 1–2 residents on duty on 24 h shifts as defined in the rest of the hospitals of the whole country as well. Divided in specialized Units, a Colorectal and an Acute Care Surgery Unit (ACSu) share responsibilities of AD healthcare, the first attending to tailored decisions and surgery on the elective setting and follow-up of ambulatory NOM with or without antibiotics and the second stablishing clinical decisions and surgery of in-hospital admitted patients because of acute episodes of AD. With this mission and since its creation, in-hospital healthcare for AD was resumed on an evidence-based local protocol promoted by the ACSu, integrating staff from the Emergency Department, Intensive Care Unit (ICU), Anesthesiology, Radiology (Diagnostic and Interventional), and Surgery. Updated in December 2017, an anonymous database was prospectively maintained for six years (January 2018–December 2023), including all patients with AD requiring hospitalization (Figure 1). After the publication of the updated WSES guidelines, this protocol was also completely adopted, and a chronological comparison was designed between two groups: Group 1, prior to the publication of the WSES guidelines (2018–2019), and Group 2, after the publication of it (2020–2023). Therefore, this is a retrospective observational study based on a prospectively maintained institutional registry, which includes all patients with acute diverticulitis who satisfied criteria for hospital admission. The registry was implemented as part of the hospital’s routine clinical documentation and quality control program. The database collected demographics, comorbidity (Charlson Comorbidity Index, CCI), clinical presentation, diagnostic findings, disease progression, perioperative and postoperative surgical details, and outcomes.

Patients were classified using the WSES system, based on radiological findings. Empirical antibiotic treatment was defined according to local hospital guidelines.

Inclusion criteria for the study were defined as follows: patients over 14 years of age who were admitted for left-sided acute diverticulitis, radiologically classified according to the World Society of Emergency Surgery (WSES) grading system. Patients managed either conservatively or surgically were included, provided that hospitalization was indicated based on the hospital’s internal protocol.

Exclusion criteria were defined as follows: patients under 14, pregnancy, patients with AD and preoperative diagnosis of colovesical or colovaginal fistula (due to their special clinical conditions and surgical implications), right colon AD, and other final diagnosis or pathologic exam after surgery different than AD. Patients with previously unsuspected intraoperative findings of minimal colovesical or colovaginal fistula were not excluded. As well, patients with AD treated on an out-patient basis were out of the scope of the present study.

We applied uniform inclusion criteria across both cohorts to minimize selection bias. All patients were classified using the same WSES staging system, reducing classification bias. Data were collected from a prospective institutional registry, and outcomes were obtained through active follow-up via medical records and patient contact, limiting information and follow-up bias.

The primary outcome indicator was PA rates. Other outcomes were NOM, the use and outcomes of damage control surgery, and the morbidity and mortality rate associated with Hartmann’s procedure (HP). NOM was defined as the initial therapeutic approach consisting of supportive care, intravenous fluid therapy, and empirical antibiotic administration according to local microbiological protocols. NOM was considered successful if patients recovered without requiring surgical intervention during hospitalization. Conversely, NOM failure was defined as the need for surgical intervention due to clinical deterioration despite appropriate antibiotic therapy or in-hospital death directly attributable to the diverticulitis episode. NOM was only evaluated in patients classified as WSES stages 0 to IIB, as patients with stages III and IV were managed surgically from the outset, according to protocol. Patients who underwent SR during admission after successful NOM because of clinical history of continuous episodes or recurrent symptoms and those who needed percutaneous drainage of abscesses but were not operated afterwards were not taken into account for defining NOM failure. PA rates included all patients undergoing colorectal anastomosis after open, laparoscopic, or DCS SR, with or without PI. Anastomotic leaks (AL) were confirmed through CT. Perianastomotic intraabdominal abscesses were included for AL rate calculation, but distant intraabdominal abscesses only counted as Organ/Space Surgical Site Infection Event (as indicated by the Center for Diseases Control and Prevention Guidelines) and were included in global morbidity [10]. Global and SR-related morbidity was stratified and described according to the Clavien–Dindo (CD) classification [11]. For better analysis, groups 0 and IA were combined, as the NOM failure rate is low in both groups. Patients with associated abscesses (IB and IIA) were also grouped together, considering the specific implications of large abscesses ≥4 cm. Stage IIB was analyzed separately, as NOM remains a subject of debate in this stage despite published results indicating a low failure rate with this approach. Finally, stages III and IV were grouped together, as they are uniformly considered indications for immediate surgery.

Patient follow-up after discharge is determined by whether the patient underwent surgery. If the patient was not operated on during hospitalization, a colonoscopy is requested upon discharge due to the risk of occult neoplasia in patients with AD stage IA or higher (above all in patients with associated abscess). This colonoscopy is recommended to be performed 4–6 weeks after discharge. After the colonoscopy, the patient is reviewed in the outpatient clinic, and treatment and follow-up are adjusted according to symptoms. If the patient underwent surgery, follow-up is conducted in person at the outpatient clinic approximately between the first 30 and 90 days after discharge. Routine colonoscopy is not mandatory for patients who have undergone surgery.

Continuous and qualitative variables were defined as mean (range) and percentages, respectively. Statistical tests applied were two-sided and included ANOVA/Kruskal–Wallis and χ2/Fisher’s exact tests, depending on the intrinsic characteristics of the analyzed variables. For the analysis of the determinants of postoperative morbidity, a logistic regression model was built, with an appropriate 95% confidence interval (CI) and OR. Findings were considered statistically significant if *p* ≤ 0.05. Data were prospectively collected and subsequently analyzed using SPSS 22 statistical software for Windows version 11 (SPSS Inc., Chicago, IL, USA).

## 3. Results

A total of 420 patients met the inclusion criteria and were included in the prospective database during the study period, with a mean age of 62.1 years (range: 24–96), 213 (50.7%) females and 207 (49.3%) males, and a mean CCI of 2.74 (range: 0–12). The data described below are summarized in Table 1. Most patients had no previous history of AD, fewer than 10% had immunosuppressive conditions, and the leading clinical presentation pattern was inflammatory. The distribution by WSES classification after an urgent CT scan before admission was as follows: 0, 107 (25.5%); IA, 146 (34.8%); IB, 56 (13.3%); IIA, 44 (10.5%); IIB, 28 (6.7%); III, 15 (3.6%); IV, 24 (5.7%). With a mean C-reactive protein (CRP) level of 114.5, a statistically significant increase was observed with increasing radiological severity of AD (*p* < 0.00). A significant association between immunosuppression status and WSES stage was observed (immunosuppressed patients: 0−IIA, 28/353, 7,36%; IIB−IV, 11/67, 14,1%; *p* < 0.05, OR 2).

The sample of early-stage acute diverticulitis (0–IA) included 253 patients. The failure rate of non-operative management (NOM) in this group was low (2.7%), although percutaneous drainage (PD) was required during the episode in seven patients. Only 19 patients underwent sigmoid resection (SR), 73.7% with a laparoscopic approach—7 due to NOM failure and 12 for recurrent episodes or persistent symptoms affecting quality of life. Primary anastomosis (PA) was the preferred option after SR in this group, as only one patient, who was under immunosuppressive therapy following a kidney transplant, required an urgent Hartmann’s procedure (HP) due to NOM failure with hemodynamic instability.

A total of 100 patients presented with a concomitant abscess (IB-IIA). PD was performed in 27 patients—4 in the IB group and 23 in the IIA group. All percutaneously accessible abscesses ≥ 4 cm were drained. In the isolated IIA group, no correlation was observed between the absence of a PD window and the need for surgery (*p* < 0.2). The overall NOM failure rate in the IB-IIA group was 16%. Surgery was performed in 27 patients in this group, 66.6% laparoscopically, with PA without PI in 23.

One patient underwent a DCS strategy (Table 2), as NOM failure presented with an extremely severe inflammatory response and septic shock; an anastomosis was performed during the second look. AL in this group occurred in three patients, one of whom required surgical intervention due to complete anastomotic dehiscence, necessitating HP.

A total of 28 patients were grouped in IIB and 39 in III–IV stages. In stage IIB, only two patients underwent SR—one with PA without PI after a laparoscopic SR and one with an open HP due to severe comorbidity. Of the 39 patients in the III–IV group, 2 were excluded from surgery due to extreme age and frailty, with a limitation of therapeutic effort, and 5 patients were managed with antibiotics and an expectant approach by the emergency surgical teams due to minimal clinical and radiological findings. The remaining 32 patients underwent SR (laparoscopically in 4 cases), with PA in 20 (only 1 requiring PI), HP in 8, and a DCS strategy with an abbreviated SR and temporary abdominal closure in 4 (Table 2). Of these four patients, three were subsequently anastomosed in later surgical revisions (one with PI), while the remaining patient ultimately underwent HP. AL occurred in six patients in the III–IV group, four managed medically or endoscopically, and two requiring surgical intervention (one converted to HP, one treated with drainage and PI). No postoperative mortality was observed in this group.

Overall, SR was performed in 20.2% of patients, with 43.5% of these procedures initiated laparoscopically and a conversion rate of 10.8%. Although not statistically significant, a progressive increase in laparoscopic procedures was observed during the study period. A global stoma-free rate of 78,8% after SR was achieved at the end of the study, with an AL rate of 15.7% and significant morbidity and mortality (CD3-5) of 14.2% overall and 17.6% when specifically considering SR-related cases.

In the comparative analysis, no significant differences were observed between the groups in terms of age, gender, Charlson Index, C-reactive protein (CRP) levels, number of surgical procedures, or percentage of immunosuppressed patients (Table 3).

Although the overall surgical rate was slightly lower in the study group after the publication of the WSES guidelines (23.3% vs. 16%), the proportion of anastomosis without protective ileostomy remained high in both groups (75.6% vs. 74.4% respectively), reflecting an already-high institutional adherence to primary anastomosis as the preferred surgical approach.

The frequency of HP decreased over time, with a complete absence in 2023, although its overall use did not differ significantly between groups (seven patients per group; *p* = 1.000). An increase in the use of the HP was observed in 2021. This rise coincided with the impact of the COVID-19 pandemic. In this context, it is plausible that, due to increased healthcare pressure, HP was favored as the preferred surgical option following sigmoid resection.

The use of damage control surgery (DCS) was comparable between groups (Group 1 *n* = 2 vs. Group 2 *n* = 3; *p* = 0.731). Its application following the WSES 2020 update contributes to a higher final rate of anastomosis, although this difference was not statistically significant (75,6% vs. 82.05%). Only one patient underwent anastomosis with ileostomy, and this occurred in Group 1 before the publication of the WSES guidelines. Notably, the laparoscopic approach was more frequently used in the study group after the publication of the WSES guidelines (29.27% vs. 46.15%), although this difference did not reach statistical significance (*p* = 0.184). When stratified by WSES grade, laparoscopic surgery was more common in lower stages (WSES 0–2), consistent with guideline recommendations. However, in line with the 2020 WSES update, which endorses laparoscopic management in experienced centers, even in advanced stages, a several number of laparoscopic procedures have been performed in advantages stages (III and above), particularly in Group 2. While the implementation of the WSES 2020 guidelines did not drastically modify the choice of surgical technique in our setting—likely due to pre-existing adherence—the refinement of protocols, increased use of laparoscopy, stronger protocolization, and improved case selection may have contributed to the observed reduction in overall morbidity (21.92% vs. 12.7%). A multivariate logistic regression model was developed to identify factors associated with the choice between primary anastomosis (PA) and Hartmann’s procedure (HP). The model included age, Charlson index, WSES grade, immunosuppression, study group, and morbidity (Clavien-Dindo ≥ I). The analysis revealed a non-significant trend suggesting that older patients were more likely to undergo a HP. There was also a trend suggesting that patients undergoing anastomosis had a lower risk of postoperative morbidity compared to those treated with HP, representing a 78% risk reduction (OR = 0.22, *p* = 0.088). The regression analysis identified the WSES classification as the only independent predictor significantly associated with the surgical technique. Higher WSES grades were linked to a lower likelihood of performing a primary anastomosis and a greater tendency toward selecting the Hartmann procedure (Table 4).

## 4. Discussion

Our study demonstrates that current evidence-based recommendations for AD, as summarized in clinical guidelines, must be strictly followed because they are effective [1,2]. Excluding peritoneal lavage, which is not considered a suitable surgical option (LOLA arm from the LADIES trial), the standard approach when surgery is required for AD, regardless of stage, is SR. After SR, the surgeon must decide between PA, with or without PI, or HP. Several observational studies have supported PA following SR, and the results of two recent RCTs (DIVERTI and the DIVA arm of the LADIES trial) have reinforced this approach. Both studies concluded that morbidity and mortality rates for the index procedure are similar between PA and HP, but the stoma-reversal rate and its associated morbidity and mortality, as well as incisional and parastomal hernias, remain concerns with HP [12,13].

Systematic reviews and meta-analyses of these studies have confirmed these findings, suggesting that overall mortality and major complications are significantly reduced with PA following SR, after both the initial operation (RR 0.67, *p* < 0.26) and the restorative procedure (RR 0.48, *p* < 0.03), with a combined analysis showing an even stronger effect (RR 0.67, *p* < 0.005) [4,5,14].

Despite this evidence, large series continue to show that the most frequently preferred option among on-duty surgeons after SR is HP. Data from the American College of Surgeons National Surgical Quality Improvement Program Colectomy Procedure Targeted Database, the Italian Society of Colorectal Surgery, and the Goodbye Hartmann multicenter trial [7,8,15] clearly indicate that evidence-based principles are not yet consistently adopted in clinical practice, as many surgeons remain reluctant to perform PA. However, our study suggests that high PA and final stoma-free rates can be achieved without increasing morbidity and mortality, provided there is an adequate selection of patients and, most importantly, a strong reliance on previously published literature. Moreover, regarding the postoperative outcomes of our series of patients, multivariate analysis showed that patients who underwent primary anastomosis without a protective ileostomy exhibited a clear trend toward lower morbidity compared to those treated with a Hartmann’s procedure.

Our study supports PA after SR without PI. Previous studies have not shown significant differences in morbidity and mortality between PA with or without PI. However, given the limited accuracy of current scoring systems in predicting AL [16], we agree that the decision to perform a PI following PA after SR should be left to the surgeon’s discretion. The results of the DIVERTI 2 trial will likely provide further insights into the role of PI following SR and PA in AD [17].

With advancements in technology and surgical expertise, laparoscopic and minimally invasive procedures have been progressively incorporated into all surgical disciplines. In accordance with this trend, studies support laparoscopic SR in AD [1,2]. This trend is reflected in our study, with the rate of laparoscopic SR performed aligning with those reported in the literature [7,8,15].

An increasing number of studies support the application of DCS principles to WSES III–IV stages of AD. Recent systematic reviews and meta-analyses have grouped these reports and shown that, following initial source control and ICU recovery of septic status, PA can be achieved in 50–75% of cases during subsequent surgical revisions. Among these, only 7–15% require PI, with an anastomotic leak rate of 7–13% and an overall mortality rate of less than 10% [6,18,19]. However, these studies include heterogeneous indications for DCS in AD, and while they represent the best available evidence to date, further clarity is expected from the results of the COOL trial [20,21]. The application of DCS principles to acute peritonitis caused by AD is a key element of our protocol. This approach resulted in an increased final anastomosis rate in our Case group, although this difference was not statistically significant. These findings support the role of DCS as an effective surgical strategy in selected clinical scenarios (Table 2). Our study supports previously published findings regarding immunosuppression as a risk factor for more severe AD. Additionally, it confirms that IIA AD with abscesses not accessible to PD can initially be treated with antibiotics alone. We also observed that a previous history of AD is not necessarily associated with a more aggressive recurrence [1,2]. Regarding NOM for WSES IIB AD, our previously published experience [22], along with more recent studies [23] and systematic reviews [24,25], has established that AD with extraluminal air—both pericolic and distant—can be successfully treated with NOM, achieving an overall success rate of 85–95%, provided there is no clinical diffuse peritonitis or hemodynamic instability.

Surgeons from various disciplines may be on duty, and like the rest of the staff in involved departments, they must be well-versed in the management of AD patients. These considerations have been integrated into a locally adapted multidisciplinary protocol, promoted and updated by the ACSu, which serves as a central pillar in guiding healthcare management for AD (Figure 1).

## 5. Limitations

The study presents certain limitations. It was conducted in a single centre, and although the *n* is large, this may limit the external validity of the results. The analysis is non-randomized, which could introduce selection or interpretation bias.

## Figures and Tables

**Table 1:** Demographics, comorbidity, clinical presentation, NOM data, peri/postoperative variables, and in-hospital stay summarized by groups.

		0–IA		IB–IIA		IIB		III–IV		Overall	
**N**		253 (60.2%)		100 (23.8%)		28 (6.6%)		39 (9.4%)		420	
**Age**		62.7 (25−96)		62.3 (25−93)		59.1 (36−90)		60.3 (24−96)		62.1 (24−96)	
**Gender (Male, yes)**		114 (45.1%)		51 (51%)		21 (75%)		21 (53.8%)		207 (49.3%)	
**CCI**		2.87 (0−10)		2.44 (0−9)		2.54 (0−12)		2.82 (0−10)		2.74 (0−12)	
**Immunosuppression (yes)**		22 (8.7%)		6 (6%)		5 (17.8%)		6 (15.4%)		39 (9.3%)	
**Pattern of presentation** **(Inflammatory, yes)**		247 (97.6%)		92 (92%)		27 (96.4%)		37 (94.9%)		403 (96%)	
**Previous episodes**	**None**	176 (69.6%)		68 (68%)		24 (85.7%)		36 (92.3%)		304 (72.4%)	
	**1**	43 (17%)		19 (19%)		1 (3.6%)		3 (7.7%)		66 (15.7%)	
	**≥2**	34 (13.4%)		13 (13%)		3 (10.7%)		0 (0%)		50 (11.9%)	
**CRP**		95 (3−488)		128 (7−387)		155 (3−505)		173 (3−636)		114.5 (3−636)	
**PD (yes)**		7 (2.7%)		27 (27%)		4 (14.3%)		-		38 (9%)	
**SR (yes)**		19 (7.5%)		27 (27%)		2 (7.1%)		37 (94.9%)		85 (20.2%)	
**NOM failure (yes)**		7 (2.7%)		16 (16%)		2 (7.1%)		Not applicable		25/381 (6.5%)	
**SR,** **additional surgical procedure (yes)**	**Gynecological**	2	6 (4 pts/19, 21%)	1	7 (5 pts/27, 18.5%)	-	-	2	8 (6 pts/37, 16.2%)	5	21 (15 pts/85, 17.6%)
	**Intestinal**	2		3		-		2		7	
	**Spleen**	-		1		-		1		2	
	**Bladder**	2		1		-		1		4	
	**Abdominal wall**	-		1		-		2		3	
**SR,** **laparoscopy (yes)/** **conversion (yes)**		14/19 (73.7%)/ 2/14 (14.3%)		18/27 (66.6%)/ 2/18 (11.1%)		1/2 (50%)/ -		4/32(12,5%)/ -		37/85 (43.5%)/ 4/37 (10.8%)	
SR, PA * (yes)		18/19 (94.7%)		22/27 (81,5%)		1/2 (50%)		21/32 (65,6%)		62/80 (77,5%)	
**SR, DCS (yes)**		-		1		-		4		5/80 (5.9%)	
**SR, AL (yes)**		2/18 (11.1%)		3/23 (13%)		-		6/28 (21.4%)		11/70 (15.7%)	
**Morbidity,** **global episode**	**None**	224/253 (88.6%)		59/100 (59%)		17/28 (60.7%)		18/39 (46.1%)		318/420 (75.7%)	
	**I–II**	15/253 (5.9%)		11/100 (11%)		4/28 (14.3%)		12/39 (30.8%)		42/420 (10%)	
	**III–IV**	8/253 (3.2%)		26/100 (26%)		6/28 (21.4%)		7/39 (18%)		47/420 (11.2%)	
	**V**	6/253 (2.3%)		4/100 (4%)		1/28 (3.6%)		2/39 (5.1%)		11/420 (3%)	
**Morbidity,** **SR specific**	**None**	11/19 (57.9%)		14/27 (51.9%)		1/2 (50%)		18/32(56,3%)		44/80 (55%)	
	**I–II**	5/19 (26.3%)		8/27 (29.6%)		1/2 (50%)		12/37,5%)		26/80 (32,5%)	
	**III–IV**	-		3/27 (11.1%)		-		7/32 (21,9%)		10/80 (12,5%)	
	**V**	3/19 (15.7%)		2/27 (7.4%)		-		-		5/80 (6,3%)	
**In-Hospital stay**		7.7 (3−40)		13 (4−23)		13.7 (5−27)		15.7 (1−49)		12.5 (3−49)	
Final status **	**Stoma-free** **(PA without PI)**	18/19		23/27		01/02/25		23/32		65/80 (81,3%)	
	**PA with PI**	-		-		-		01//32		1/80 (0,8%)	
	**HP**	01//19		4//27		01/02/25		8//32		14/80 (17.5%)	

CCI: Charlson Comorbidity Index. CRP: C-Reactive Protein. PD: Percutaneous Drainage (during episode or before surgery). NOM: Non-Operative Management. SR: Sigmoid Resection. Gynecological: ingle or double anexectomy and/or hysterectomy. Intestinal: mall bowel suture and/or resection and/or appendectomy. Spleen: hemostatic procedures and/or splenectomy. Bladder: cystorraphy. Abdominal wall: hernia repair and/or on-lay mesh with or without anterior components separation. DCS: Damage Control Surgery. AL: Anastomotic Leak. *: includes PA final status after DCS. **: calculated for operated patients alive at the end of the episode.

**Table 2:** Patients with a Damage Control Surgery strategy: demographics, WSES classification, reasons to adopt a DCS strategy, perioperative details, and final status.

Patients	1	2	3	4	5
**Age**	63	41	63	74	50
**Gender**	Male	Male	Male	Female	Male
**WSES classification**	IV	III	IV	III	IIA
**DCS indications**	Haemodynamic instability, inotropic support, kidney failure (C 2.2 mg/dl), purulent (4 quadrants) and faecaloid (left iliac fossa and Douglas) peritonitis, metabolic acidosis	Haemodynamic instability with inotropics, kidney failure (C 2.5 mg/dl), severe purulent peritonitis, pH 7.12, INR 1.6, lactate 2.6 mmol/L	Septic shock with NA-dependent hypotension, kidney failure (C 2.4 mg/dl), leukopenia (2600/µL), diffuse purulent and faecaloid peritonitis	High inotropic support, kidney failure (C 4.4 mg/dl), pH 7.01, lactate 3.8 mmol/L, bicarbonate 13.4 mEq/L)	High-demanding inotropic support (NA 11 ml/h), severe faecaloid peritonitis
**Initial procedure**	Abbreviated SR, free stumps, vacuum-assisted commercial TAC	Abbreviated SR, free stumps, vacuum-assisted commercial TAC,	Abbreviated SR and small-bowel resection, free stumps, vacuum-assisted commercial TAC	Abbreviated SR and left anexectomy, free stumps, vacuum-assisted commercial TAC	Abbreviated SR, free stumps, vacuum-assisted commercial TAC
**Number of revisions**	1	3	1	2	1
**Colorectal anastomosis (final)**	Yes	No	Yes	Yes (plus PI)	Yes
**AL (management)**	Yes (medical)	-	No	No	No
**Abdominal wall management**	Closed without mesh	Mesh-mediated fascial traction. Finally closed with anterior component separation and on-lay mesh	Closed without mesh	Closed without mesh	Closed without mesh
**ICU stay**	6	10	5	14	5
**Final status**	Alive, stoma-free	Alive, HP (restorative procedure 10 months later)	Alive, stoma-free	Alive, PI (restored 12 months later)	Alive, stoma-free

WSES: World Society of Emergency Surgery. DCS: Damage Control Surgery. AL: Anastomotic Leak. ICU: Intensive Care Unit. C: Creatinine. NA: Noradrenaline. SR: Sigmoid Resection. TAC: Temporary Abdominal Closure. INR: International Normalized Ratio. PI: Defunctioning proximal ileostomy.

**Table 3:** Demographics, comorbidity, and perioperative variables in both comparison groups.

	Group Before WSES Guidelines *n* = 176	Group After WSES Guidelines *n* = 244	*p* < 0.05
**Age (median)**	64 (28–96)	62 (24–96)	N.S.
**Charlson Index (median)**	3 (0–10)	2 (0–59)	N.S.
**CRP (median)**	107 (0–636)	95 (0–477)	N.S.
**Number of surgeries**	41/176 (23.3%)	39/244 (16%)	N.S.
**mmunosuppression**	21/176 (11.9%)	18/244 (7.4%)	N.S.
**Men (%)**	91/176 (51.7%)	116/244 (47.5%)	N.S.
**PA stoma-free**	31/41 (75.6%)	29/39 (74.4%)	N.S.
**HP**	7/41 (17.9%)	7/39 (17.07%)	N.S.
**DCS**	2/41 (4.9%)	3/39 (5.1%)	N.S
**Final Status DCS, anastomosis stoma-free**	0/5 (DCS)	3/5(DCS)	N.S.
**Total anastomosis stoma-free**	31/41 (75.6%)	32/39 (82.05%)	N.S.
**Laparoscopy**	12/41 (29.7%)	18/39 (46.1%)	N.S.
**MORBIDITY**	**37/176 (21.92%)**	**21/244 (12.7%)**	**0.03**
**Major Surgical Morbidity**	6/41 (14.6%)	6/39 (15.4%)	N.S.

CRP: c-reactive protein; PA: primary anastomosis; HP: Hartmann procedure; DCS: Damage Control Surgery. N.S: no significance.

**Table 4:** Logistic regression. Factors associated with the choice of non-ileostomy anastomosis vs. Hartmann’s.

Variables	OR (IC 95%)	*p*
**Groups (before vs. After WSES guideline)**	1.85 (0.36–9.47)	0.46
**Age**	**0.95 (0.89–1.01)**	**0.09**
**Charlson**	0.94 (0.60–1.47)	0.77
**WSES STAGE**	0.64 (0.43–0.96)	0.03
**Immunosuppression**	0.29 (0.03–2.60)	0.27
**Morbidity**	**0.22 (0.04–1.25)**	**0.09**

WSES: World Society of Emergency Surgery.

## Data Availability

During the preparation of this manuscript/study, the author used ChatGPT version 4o for the purposes of generating graphs. The authors have reviewed and edited the output and take full responsibility for the content of this publication.

## Abbreviations

AD	Acute Diverticulitis
CT	Computed Tomography
NOM	Non-Operative Management
PD	Percutaneous Drainage
SR	Sigmoid Resection
PI	Defunctioning Proximal Ileostomy
HP	Hartmann’s Procedure
DCS	Damage Control Surgery
TAC	Temporary Abdominal Closure
ACSu	Acute Care Surgery Unit
ICU	Intensive Care Unit
CCI	Charlson Comorbidity Index
WSES	World Society of Emergency Surgery
ASCRS	American Society of Colon & Rectal Surgeons
PA	Primary Anastomosis
AL	Anastomotic leak
CD	Clavien–Dindo classification of morbidity and mortality
CRP	C-Reactive Protein
RCT	Randomized Clinical Trial
